# Optimizing the Ovarian Tissue Cryopreservation in the ‘Oncofertility’ Institutional Program at an Italian National Cancer Institute

**DOI:** 10.3390/healthcare11202727

**Published:** 2023-10-13

**Authors:** Erica Silvestris, Carla Minoia, Giuseppe De Palma, Ondina Popescu, Anna Altavilla, Attilio Guarini, Fabio Pavone, Vera Loizzi, Gennaro Cormio, Raffaella Depalo

**Affiliations:** 1 Gynecologic Oncology Unit, IRCCS Istituto Tumori “Giovanni Paolo II” Bari, 70124 Bari, Italy; vera.loizzi@uniba.it (V.L.); gennaro.cormio@uniba.it (G.C.); 2 Hematology Unit, IRCCS Istituto Tumori “Giovanni Paolo II” Bari, 70124 Bari, Italy; c.minoia@oncologico.bari.it (C.M.); attilioguarini@oncologico.bari.it (A.G.); f.pavone@oncologico.bari.it (F.P.); 3 Institutional BioBank, Experimental Oncology and Biobank Management Unit, IRCCS Istituto Tumori “Giovanni Paolo II” Bari, 70124 Bari, Italy; r.depalo@oncologico.bari.it; 4 Pathological Anatomy Unit, IRCCS Istituto Tumori “Giovanni Paolo II” Bari, 70124 Bari, Italy; o.popescu@oncologico.bari.it (O.P.); a.altavilla@oncologico.bari.it (A.A.); 5 Department of Interdisciplinary Medicine (DIM), University of Bari “Aldo Moro”, 70121 Bari, Italy

**Keywords:** cancer treatment-related infertility (CTRI), fertility preservation, gonadotoxicity, ovarian tissue cryopreservation, slow freezing (SF), ultra-rapid freezing (URF)

## Abstract

Background: The majority of female cancer patients undergoing anticancer treatments are at risk of experiencing ‘cancer treatment-related infertility’, which can result in permanent damage to their reproductive prospects. Among the fertility preservation methods, ovarian tissue cryopreservation (OTC) has emerged as an alternative for these patients. The Cancer Institute of Bari initiated a research program to assess the feasibility of OTC. This study compares the viability of ovarian cortical fragments cryopreserved using slow freezing (SF) and ultra-rapid freezing (URF) methods. Methods: Ovarian cortex biopsies were obtained from 11 fertile women enrolled in our oncofertility service between June 2022 and January 2023. After tissue collection, a histological assessment was performed before cryopreservation. OTC was carried out using both SF and URF methods. Six months later, thawed samples were evaluated for follicle counts and histological integrity. Results: No statistically significant difference was observed in the proportion of intact follicles (means of 31.5% and 73.0% in the SF and URF groups, respectively; *p* = 0.064). However, there was a significant difference in the number of follicles between the SF group (n = 149) and the URF group (n = 37) (*p* = 0.046). Conclusions: We assessed the viability of ovarian cortex after freezing and thawing, focusing on the structural integrity of follicles. Our findings suggest that there are no significant differences between the SF and URF methods.

## 1. Introduction

The majority of female cancer patients who require anticancer treatments are at risk of developing ‘cancer treatment-related infertility’ (CTRI) due to the gonadotoxic effects of these therapies. This can lead to permanent damage to their reproductive potential. Addressing the issue of CTRI is crucial, as young female cancer patients often experience significant psychological and emotional distress when their fertility is compromised, affecting their sense of identity as women. Therefore, the field of oncofertility has emerged in clinical oncology to provide education and counseling to female patients facing cancer treatments. A multidisciplinary approach involving medical oncologists, surgeons, psychologists, endocrinologists, and gynecologists/oncologists is essential to develop suitable oncofertility programs based on factors such as age, ovarian reserve, cancer type, treatment urgency, and therapy options [1,2]. In addition to the commonly used procedures of oocyte and embryo cryopreservation for fertility preservation (FP) in female patients, the early cryopreservation of ovarian cortex fragments has been proposed as an alternative method. This approach offers several advantages, including the ability to retrieve immature eggs without the need for hormonal stimulation, which is typically required for conventional ovulation induction and oocyte retrieval. Furthermore, ovarian cortex biopsies can be obtained quickly via laparoscopy, making this procedure suitable for prepubertal girls and adult patients with hormone-sensitive cancers who require urgent neoadjuvant anticancer therapies, such as chemotherapy or radiation treatments [3,4,5]. Regarding the success of ovarian cortex reimplantation (OCR) in achieving pregnancies, the first live birth after the reimplantation of autologous cryopreserved ovarian cortex using the slow freezing (SF) technique was reported in 2003 [1,6]. Subsequent clinical studies supported the effectiveness of this method in achieving successful pregnancies and live births (LBs). As a result, the American Society for Reproductive Medicine (ASRM) recognized autologous ovarian cortex reimplantation as a safe and clinically acceptable FP procedure for various conditions, including hematologic malignancies, neurological tumors, sarcomas, and non-malignant diseases [7,8,9]. However, it has been discouraged in certain hematologic malignancies, particularly leukemia, due to the potential risk of reintroducing leukemic cells from the cryopreserved ovarian tissue [10]. Autologous ovarian tissue transplantation aligns with European regulations for ensuring the quality and safety of cryopreserved tissue [11,12,13]. The European Union Council has issued directives and guidelines to promote tissue and organ donation for transplantation while safeguarding public health and recipient safety. This includes accreditation requirements for establishments involved in storing, preparing, and distributing tissues and cells, as well as training for personnel [14]. Furthermore, the European Society for Human Reproduction and Embryology (ESHRE) provides specific guidelines for assisted reproduction procedures that align with common regulations in European Union countries, including the cryostorage of eggs and ovaries [14]. In Italy, guidelines established by Law 40/2004 “Regulations on Medically Assisted Procreation (PMA)” contain recommendations for the safe freezing of reproductive cells and tissues, along with regulations defining quality and safety standards for the donation, recovery, freezing, and utilization of human cells and tissues [14]. Based on these principles, the Cancer Institute of Bari (IRCCS Istituto Tumori “Giovanni Paolo II” Bari) initiated a research program funded by the Italian Ministry of Health [14]. The program aims to implement ovarian tissue cryopreservation (OTC) for female cancer patients undergoing anticancer treatments with the goal of establishing an oncofertility reference center for FP. This center would also serve other cancer centers within and beyond the region. This pilot study seeks to compare the effects of cryopreservation using the slow freezing (SF) and ultra-rapid freezing (URF) methods for human ovarian cortical tissue and to evaluate their potential for establishing an elective OTC program at our cancer institute.

## 2. Materials and Methods

Steps in the OTC program:

### 2.1. Implementing a Working Team for Counselling Patients and Ovarian Cortex Sampling

In November 2014, the National Transplantation Center of the Italian Ministry of Health issued guidelines for the establishment of cryobiological laboratories at tissue institutions. These guidelines defined structural requirements and safety standards for biobanks, including specific requirements for handling samples from virus-infected patients. Additionally, the Apulia region established regulations for in vitro fertilization (IVF) centers, stipulating that procedures for collecting and storing ovarian cortex should be carried out in third-level IVF centers with surgical expertise and personnel skilled in embryology and laboratory work [1]. To provide fertility preservation services for young cancer patients undergoing chemotherapy and/or radiotherapy, IRCCS Istituto Tumori “Giovanni Paolo II” Bari initiated a program in 2019. This program, located at the regional oncologic hub center, involved preliminary clinical evaluations by an interdisciplinary counseling team, which included specialized oncologists and fertility experts. Patients were assessed based on cancer type and stage to determine the most suitable FP procedure (e.g., oocyte or tissue cryopreservation, or gonadal shielding with gonadotropin-releasing hormone analogs). Ovarian reserve was evaluated using anti-Müllerian hormone (AMH) levels and the ultrasound antral follicle count (AFC). Psychologists provided counseling throughout the process. OTC was particularly beneficial for young patients with hormone-sensitive cancers, as it allowed for immediate anticancer treatment without delaying for ovarian stimulation [1]. Several factors influenced the decision to offer OTC, including the patient’s age and expected 5-year survival rate, the absence of metastases, and the absence of contraindications for concurrent surgery [1].

### 2.2. Patients’ Characteristics and Enrollment Criteria

As part of our research project, we collected ovarian cortex biopsies from 11 fertile women who were enrolled in our oncofertility outpatient clinic at the IRCCS Istituto Tumori “Giovanni Paolo II” Bari between June 2022 and January 2023. These patients were 28–34 years old (average age: 31) and had benign gynecologic diseases or early-stage urogynecologic malignancies (excluding ovarian tumors). They were scheduled for abdominal/pelvic surgery and had 5-year survival probabilities exceeding 50%. The removal of an ovarian cortex fragment did not impact disease staging or prognosis in any case. The enrollment criteria included serum AMH levels ≥ 2 ng/mL and AFC ≥ 8 [15] (Table 1). The exclusion criteria comprised metastatic cancer, prior chemotherapy or immunotherapy treatments, premature ovarian failure, and positive serological tests for HBV, HCV, or HIV. This study was approved by the Ethical Committee of the IRCCS Istituto Tumori “Giovanni Paolo II” Bari (doc. 616/2020), and all enrolled patients provided written informed consent to donate ovarian tissue solely for research purposes.

### 2.3. Ovarian Cortex Recruitment and Sampling

Ovarian cortex biopsies were primarily obtained via minimally invasive laparoscopy for patients scheduled for abdominal/pelvic surgery. In rare instances, laparotomy was required for specific cases. The collected tissue fragments, approximately 1 cm × 4–5 mm × 1–1.5 mm in size, were promptly placed in Leibovitz’s L-15 medium containing Glutamax (Gibco, Life Technologies, Bleiswijk, The Netherlands) [1] and quickly transported to the Institutional Biobank laboratory. The cortex component was carefully separated from the medulla and dissected into thin strips (2 × 6 mm with 1 mm thickness) in a biological safety cabinet. A portion of the collected and dissected tissue was sent to the Pathological Anatomy Unit laboratory for histopathological examination.

### 2.4. Ovarian Cortex Cryopreservation Procedure: Slow Freezing vs. Ultra-Rapid Freezing

The ovarian cortex strips were individually placed in pre-cooled sterile cryovials (Biosigma, Cona, Italy) containing 1 mL of a cryoprotective solution composed of Leibovitz’s L-15 medium supplemented with 4 mg/mL human serum albumin and 1.5 mmol/L DMSO, following the procedure described by Donnez et al. [1]. The samples were randomly assigned to either the SF [1] or ultra-rapid freezing (URF) [16] groups. SF involved using a programmable freezer (Kryo 560-16; Planer Limited, Sunbury-On-Thames, UK). Cryovials were initially cooled to 0 °C for 15 min, followed by freezing at 2 °C/min until −7 °C. Seeding was manually initiated at this temperature, followed by freezing at 0.3 °C/min to −40 °C and further freezing at −10 °C/min until −140 °C. Finally, the slices were submerged into liquid nitrogen using lower cryoprotectant concentrations compared to URF [1]. URF, on the other hand, is a faster cryopreservation procedure that involves rapid immersion of the cryovial containing the ovarian cortex tissue in liquid nitrogen. All samples were cryopreserved for six months, after which they were thawed in a 37 °C water bath for 2 min. The tissue fragments were then isolated from the cryoprotective solution and washed three times with fresh Leibovitz’s L-15 medium for histological evaluation.

### 2.5. Histological Evaluation

Ovarian cortical tissue includes several cell types such as stromal cells, follicles (each containing an oocyte and granulosa cells), and blood vessels. Maintaining the integrity of stroma and the vascular system is crucial for follicular development and the restoration of gonadal function after transplantation [17]. Therefore, assessing cryopreservation efficiency requires analyzing not only follicles but also other cellular components within the ovarian cortex tissue. Thus, to compare the efficiency of the SF and URF methods, we examined the quality of different elements within the ovarian cortex fragments (follicles, stromal cells, and the vascular system) before and after freezing. Frozen ovarian cortex pieces from the biobank were cut into approximately 1 cm^2^ sections with a thickness of 1 mm. These fragments were fixed overnight at 4 °C in an alcohol–formalin–acetic acid solution for histological evaluation. The fixed samples were embedded in paraffin and sliced into 4 μm serial sections. Each set of 8 consecutive sections was placed on a slide, and every second slide was deparaffinized, hydrated, and stained with hematoxylin, eosin, and saffron (HES). The HES sections were examined using light microscopy (BX40; Olympus, Tokyo, Japan) at 400× magnification. During the freezing and thawing process, tissue changes occurred at the morphological, ultrastructural, and functional levels [18]. Follicles were considered atretic if they exhibited oocytes with eosinophilic cytoplasm, chromatin material contraction, and clumping. The follicle morphology was assessed based on the Keros parameters, which define follicles as intact when no degeneration is observed in the oocyte and granulosa cells and when the oocytes remain in contact with the surrounding granulosa cells while maintaining an intact basement membrane attached to the granulosa cell layer [19]. The vascular integrity was evaluated based on the presence of an intact vascular endothelium without signs of detachment [20]. Vascular anomalies were defined as endothelial detachment, internal elastic membrane, or smooth muscle degeneration, while stromal cells were assessed for pycnotic nuclei expression (counted in 3 HPF fields according to Keros) [19,20].

### 2.6. Statistical Analysis

The statistical analysis was conducted using GraphPad Prism version 5.03 software (GraphPad Software, San Diego, CA, USA). The Kruskal–Wallis non-parametric test with the Bonferroni correction was used to analyze the data, with a *p*-value of <0.05 considered statistically significant.

## 3. Results

### Histological Evaluation of Thawed Strips 

Between June 2022 and January 2023, a total of 20 strips from ovarian cortex samples were thawed, with 11 undergoing SF and 9 undergoing URF.

**Figure 1:**
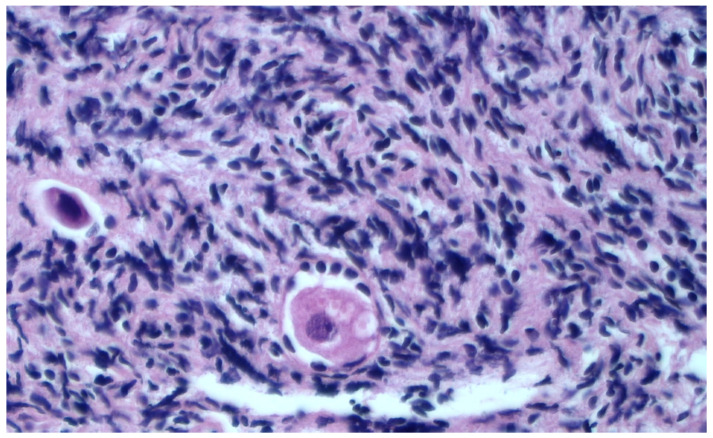
HES-stained section of a representative fresh ovarian cortex sample. Presence of crushing artifacts can be observed in the pre-freezing sample. H&E staining at 10× magnification.

To compare the efficiency of the SF and URF procedures, we initially assessed the quality of various elements within the ovarian cortex fragments (follicles, stromal cells, and the vascular system) before cryopreservation (Figure 1).

**Figure 2:**
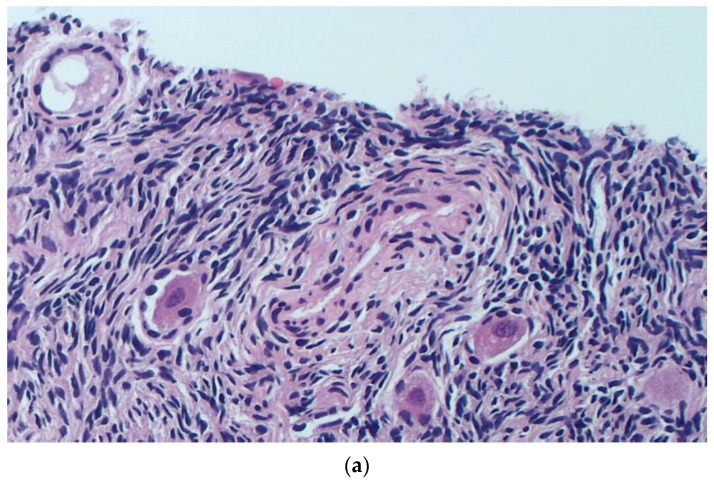

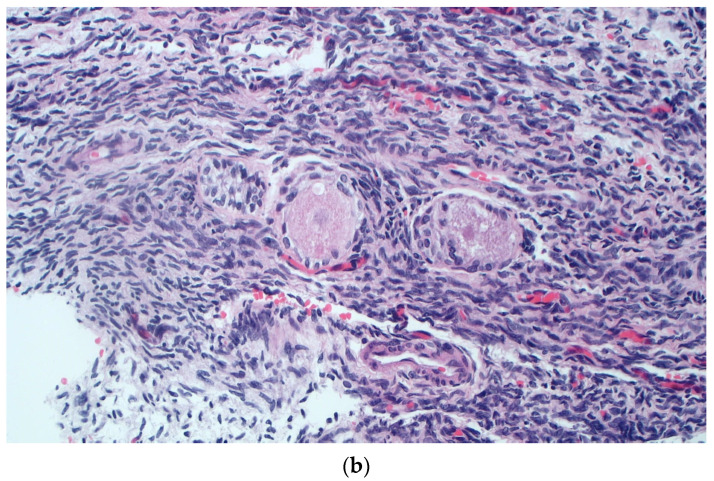
(**a**) Fragment of ovarian cortex after SF procedure. As shown, the majority of cellular structures as well as the components of follicles remained mostly intact after thawing the tissue in this sample. H&E staining at 10× magnification. (**b**) Fragment of ovarian cortex thawed after URF procedure. As shown, several morphological alterations in both stroma and vessels were present in this sample. H&E staining at 20× magnification.

After six months of cryostorage, 2 to 5 slices from each strip were subjected to a histological examination to evaluate the maintenance of the morphological aspects of the samples and perform a follicle count (Figure 2a,b).

There was no statistically significant difference in the proportion of intact follicles (means of 31.5% and 73.0% in the SF and URF groups, respectively; *p* = 0.064) (Table 2). However, a significant difference was found in the number of follicles observed in the SF group (n = 149) when compared with the URF group (n = 37) (*p* = 0.046). Table 2 summarizes the results of this morphological analysis.

## 4. Discussion

With respect to the conventional oocyte retrieval after hormone stimulation and the freezing of eggs or embryos, the OTC procedure based on the surgical recruitment of ovarian cortex strips and subsequent reimplantation in patients after cancer healing (5 years after obtaining disease remission) provides the advantage of both avoiding estrogenic stimulation and the maintenance of scheduled urgent anticancer treatments. Thus, as an FP method in oncofertility, OTC is equally effective for young female patients with hormone-sensitive cancers as for adult cancer-surviving females for the potential restoration of the endocrine balance [21,22,23]. Here, we evaluated the ovarian cortex viability after freezing and thawing, as the structural integrity of the follicles, and found that the SF procedure is equivalent to the URF technique as a minimally damaging method to cryopreserve cortical fragments.

Furthermore, based on the absence of a regional reference center for FP, we propose that in line with the oncofertility programs to be developed following the Italian Health Ministry and Italian Association of Medical Oncology guidelines, a possible implementation of the OTC procedure in young patients with hormone-sensitive cancers could strengthen the ongoing FP activities at the IRCCS Istituto Tumori “Giovanni Paolo II” Bari, which is already funded by the Ministry of Health to develop novel methodologies to prevent CTRI [14].

Our results after thawing demonstrate that follicular integrity rates are comparable between SF and URF. Indeed, stromal and vascular structures generally had minimal artifacts that did not significantly damage the follicular assembly. However, upon histological inspection, a higher number of follicles were observed in the strips cryopreserved with the SF method than in the samples subjected to the URF procedure, which was principally related to the randomization of ovarian cortex strips in the URF method group and SF method group.

The SF method is based on the gradual lowering of the cryogenic condition to reach the temperature of the liquid nitrogen. It was introduced in 1966 [24] and is commonly used for the cryostorage of ovarian tissue strips, resulting in good efficiency in successfully restoring ovarian endocrine functions several months after transplantation, namely, within five months [7]. Furthermore, in comparison to vitrification, the SF of eggs provides a higher rate of LBs [25,26] compared to vitrification, although a systematic evaluation of differences is currently unavailable [5]. In fact, only sporadic studies report an apparent superiority of the SF procedure with respect to vitrification in terms of effective pregnancies and/or LBs [7,27,28,29,30]. On the other hand, URF is a cryopreservation procedure generally adopted to freeze eggs and embryos, while is has not been properly investigated in OTC and, similar to the vitrification procedures of ovarian cortex, there are not enough data to define its suitability.

However, although OTC appears to be a preferable procedure for young patients, several aspects related to the potential risks of this procedure should be considered. In fact, a major risk is related to the possible reimplantation of malignant cells together with the ovaries, which is classified as low, medium, or high in relation to the oncological disease [31]. In fact, the major solid tumors in the early stages, such as breast cancer up to stage II and cervical carcinoma as well as Hodgkin lymphoma and Wilm’s tumor are considered to have risks as low as less than 0.2% to regenerate the disease [32]. This is strictly related to the natural history of these cancers, which are normally unable to diffuse within the ovarian tissue. In contrast, the risk of replacing the original tumor in ovaries after OTC increases up to 11% in patients affected by stage IVb breast cancer, non-Hodgkin’s lymphomas, cervical carcinoma, and Ewing’s sarcoma [31,33,34], whereas it has been reported that patients with acute leukemias, Burkitt lymphoma, and neuroblastoma are at the highest risk (>11%) of tumor renewal after OTC [35,36,37]. Thus, this procedure should not be preferred, at least in young patients with a high risk of revitalization of the original tumor.

A further interest of our research group was to combine the established cryopreservation techniques of the ovarian cortex with basic research on OSCs, which is actually in progress at the IRCCS Istituto Tumori “Giovanni Paolo II” Bari. In fact, we have already published preliminary work describing the phenotypic integrity and, theoretically, the functional viability of ovarian stem cells (OSCs) after the thawing of ovarian cortex tissue and their propensity to differentiate in vitro into mature oocytes as previously described [38]. Their postulated utilization in the future for FP programs in oncofertility would definitely overcome both the risks and restrictions related to the cryopreservation of ovarian cortex to be reimplanted in patients. In fact, the application of OSCs in FP programs will functionally solve the risk of hormone stimulation to recruit oocytes to be cryopreserved.

## 5. Conclusions

The three-year project on oncofertility has allowed the activation of a multidisciplinary working group for the identification of patients who can benefit from OTC, as well as the definition of the fate and process of tissue samples, the preferable method of freezing, the functional properties of thawed cortical tissues, and the potential utilization of OSCs. The entire process led to the request for the regional certification of the hub as a center for OTC collection and storage. Our working group will continue carrying out the activities to maintain the requirements established by the legislation for OTC in an integrated program of oncofertility to be extended to external regional institutions.

## Figures and Tables

**Table 1:** Characteristics of patients enrolled in this study.

N	Age	AMH (ng/mL)	AFC	Diagnosis
1	34	2.70	8	Uterine fibromatosis
2	28	4.89	18	Left ovarian cyst
3	30	3.56	15	Uterine fibromatosis
4	29	5.45	20	Endometriosis
5	30	3.75	16	Cervical cancer
6	33	2.89	10	Uterine fibromatosis
7	31	3.56	10	Left ovarian cyst
8	32	3.01	14	Cervical cancer
9	33	2.42	9	Endometriosis
10	24	3.98	14	Right ovarian cyst
11	34	3.98	11	Cervical cancer

**Table 2:** Strip evaluation in terms of follicle count after thawing.

Techniques	No. of Follicles after Thawing	Morphological Quality Classification after Thawing
Slow freezing	149	47 intact
		102 damaged
Ultra-rapid freezing	37	27 intact
		10 damaged

## Data Availability

The data presented in this study are available on request from the corresponding authors. The data are not publicly available because are propriety of IRCCS Istituto Tumori “Giovanni Paolo II” Bari.
